# Assessing the effects of exposure to a SARS-CoV-2 re-positive patient in healthcare personnel

**DOI:** 10.1186/s13104-020-05365-y

**Published:** 2020-11-07

**Authors:** Yoshihiko Ogawa, Koji Nishida, Iwao Gohma, Kei Kasahara, Hisakazu Yano

**Affiliations:** 1 Department of Infectious Diseases, Sakai City Medical Center, Ebaraji 1-1-1, Sakai, Osaka Japan; 2 Treatment Team for COVID-19, Sakai City Medical Center, Ebaraji 1-1-1, Sakai, Osaka Japan; 3 Department of Respiratory Medicine, Sakai City Medical Center, Ebaraji 1-1-1, Sakai, Osaka Japan; 4 Center for Infectious Diseases, Nara Medical University, Shijo-cho 840, Kashihara, Nara, Japan; 5 Department of Microbiology and Infectious Diseases, Nara Medical University, Shijo-cho 840, Kashihara, Nara, Japan

**Keywords:** SARS-CoV-2, COVID-19, Healthcare personnel, PCR re-positive

## Abstract

**Objective:**

To evaluate whether patients with COVID-19 who have tested re-positive with the PCR test for the SARS-CoV-2 virus are infectious is a challenge in the current circumstances. A follow-up survey was conducted with healthcare personnel (HCP) who were exposed to a patient whose PCR test results for SARS-CoV-2 were re-positive 18 days after the initial confirmation of negative PCR results.

**Results:**

We studied a total of 15 HCP who had contact exposures (15/15) and aerosol exposures (7/15). None of them tested positive for IgG against SARS-CoV-2 on blood examination. None of them had any symptoms during 10 days of active isolation. All PCR tests conducted using the nasopharyngeal swabs collected from the HCP on day 10 were negative. No apparent infection was found in any of the HCP who had contact exposure with and/or aerosol exposure to the patient whose PCR test results for SARS-CoV-2 were re-positive 18 days after the initial confirmation of negative results of PCR tests for SARS-CoV-2.

Clinical trial: Trial Registration: No. 170, approved June 10th, 2020 by the ethics committee of Sakai City Medical Center.

## Introduction

The spread of COVID-19 is still on-going in various regions. However, a new problem in regions where the epidemic has already been recognized is that patients are testing positive with the PCR test after the post-treatment results were negative.

In our hospital, another problem regarding COVID-19 patients is that it is difficult to find a transfer destination for the patients in whom management required physical disposal of protective equipment. This primarily occurs after long-term management in intensive care units, because especially in long-term care facilities, the medical protective equipment is insufficient. Thus, we created a policy that for patients whose sputum and/or nasopharyngeal PCR test results for SARS-CoV-2 were negative when measured twice separately over 24 h, we stopped the precautions against aerosol transmission. If the PCR test results were negative again after 72 more hours, we stopped the contact precautions for COVID-19 transmission as well. This “72 more hours” is based on the report that SARS-CoV-2 has been detected for up to 72 h after application to surfaces such as plastic and stainless steel [1]. A patient (an 81-year-old woman) who met the abovementioned criteria was waiting for rehabilitation transfer; however, she presented with worsening heart failure, due to which we determined that intensive care was required. Due to the sudden change in symptoms, she underwent nasopharyngeal and sputum PCR testing for SARS-CoV-2 again; the results were re-positive. Due to this, several HCP who had interacted with this patient had to be marked for active isolation, as recommended by the CDC [2]. We evaluated whether COVID-19 infection had occurred in the HCP.

## Main Text

### Material and Methods

We identified the HCP who needed isolation in our hospital according to the CDC guidelines for asymptomatic HCP who are exposed to individuals with confirmed COVID-19 [2]. We also performed viral culture for the index patient at Nara Medical University, Nara, Japan. The patient consented to the testing. The exposed HCP themselves carried out active isolation at home, as recommended by the infection control team in our hospital. Before active isolation, qualitative tests for the COVID-19 IgG antibody from Abbott® (Abbott ARCHITECT SARS-CoV-2 IgG test, Illinois, USA) were performed for all. After isolation, the HCP self-reported their daily physical condition and body temperature; if any physical abnormality was noted, an outpatient consultation was conducted. PCR testing of a nasopharyngeal swab was performed for each HCP on the 10th day after exposure. This study was approved by the Ethical Committee of Sakai City Medical Center.

### Results

The index patient first developed COVID-19 symptoms 10 days before she was admitted to our hospital. She was diagnosed with COVID-19 using the PCR test for SARS-CoV-2 before admission. She needed ventilator oxygenation and was placed in the ICU soon after admission. During her clinical course, she was prescribed low-dose systemic steroid therapy (0.8 mg/kg/day prednisone) for organized pneumonia and tracheotomy was performed. Approximately 26, 29, and 33 days after the first symptoms appeared, nucleic acid amplification tests for the specimen from the lower respiratory tract of SARS-CoV-2 were performed; the results of all tests were negative. The patient was subsequently transferred to the general ward. On the 44th day of illness, she developed aspiration pneumonia and pulmonary edema and was placed in the ICU again. At that time (44th day of illness and 18 days after initial confirmation of negative results of PCR), the PCR tests for the specimens from the lower respiratory tract and nasopharyngeal swab were performed again, and the results were both positive (Ct values: 33.53 for the specimen from the lower respiratory tract, and 36.83 for that from the nasopharyngeal swab). We also performed viral cultures for these specimens over a week, but the cultures were negative. The result of the test for IgG antibodies to SARS-CoV-2 was positive on the 50th day of illness (titer: 9.2 s/co). We did not perform PCR tests for SARS-CoV-2 until the 63rd day of illness, and the result of this test was re-negative.

There were 15 HCP: 2 doctors, 10 nurses, one speech-language-therapist, and 2 physical therapists (Table 1). Blood examinations taken within 2 days of exposure revealed negative IgG antibodies to SARS-CoV-2 in all subjects. None of the HCP who had been under active isolation reported any physical symptoms, including fever and respiratory difficulties, during the 10-day isolation period. All PCR tests performed using a nasopharyngeal swab obtained on the 10th day after the exposure were negative, and the results of the tests for IgG antibodies to SARS-CoV-2 on the specimens collected approximately 20 days after exposure were also negative.

**Table 1:** Characteristics of the healthcare personnel and the PPE they were wearing

No	Occupation	Age	Wearing PPE	Lack of recommended protection	Exposures
1	Nurse	20′s	Gloves, surgical mask, eye protection	Gown	Contact
2	Nurse	20′s	Gloves, surgical mask, eye protection	Gown	Contact
3	ST	40′s	Gloves, surgical mask, eye protection	Gown and N95 mask	Contact and aerosol
4	Nurse	40′s	Gloves, surgical mask, eye protection	Gown	Contact
5	Nurse	30′s	Gloves, surgical mask, eye protection	Gown	Contact
6	Nurse	20′s	Gloves, surgical mask	Gown, eye protection, N 95mask	Contact and aerosol
7	Doctor	20′s	Gloves, surgical mask	Gown, eye protection	Contact
8	Doctor	40′s	Gloves, surgical mask	Gown, eye protection, N 95mask	Contact and aerosol
9	PT	20′s	Gloves, surgical mask, eye protection	Gown, N95 mask	Contact and aerosol
10	PT	30′s	Gloves, surgical mask, eye protection	Gown, N95 mask	Contact and aerosol
11	Nurse	40′s	Surgical mask	Gloves, gown, eye protection	Contact
12	Nurse	50′s	Gloves, surgical mask, eye protection	Gown , N95 mask	Contact and aerosol
13	Nurse	20′s	Gloves, surgical mask, eye protection	Gown , N95 mask	Contact and aerosol
14	Nurse	40′s	Gloves, surgical mask	Gown , eye protection	Contact
15	Nurse aid	50′s	Gloves, surgical mask, eye protection	Gown	Contact

*PPE* Personal Protective Equipment, *ST* Speech-Language-Hearing Therapist, *PT* Physical Therapist

The recommended protection was based on the guidance of Centers for Disease Control and Prevention

### Discussion

Since the first cases were reported in China in December 2019, COVID-19 has become a global pandemic. However, despite the fact that COVID-19 cases are gradually decreasing in many countries, a new problem has arisen: a certain number of patients are testing re-positive when subjected to the PCR test [3]. To date, the risk factors and clinical characteristics of patients in whom PCR for SARS-CoV-2 was re-positive are unclear [3–8]. The core issue posed by this phenomenon is determining whether the patients whose PCR test results are re-positive are infectious. Currently, the studies trying to figure out this question tend to demonstrate the low probability of infection form a re-positive case. Kang [3] stated that in 3.3% of COVID-19 cases, the results of PCR for SARS-CoV-2 were re-positive after the patients were released from quarantine, and there were no additional infection cases arising from the re-positive patients. Lu also described that 87 re-tested as SARS-CoV-2 positive in circumstances of social isolation among 619 discharged COVID-19 cases, and no infectious strain could be obtained by culture and no full-length viral genomes could be sequenced from re-positive cases [4]. Wölfel et al. described that viral cultures were negative 8 days after the onset of COVID-19 symptoms in all the patients in their study [7]. An experimental infection model in monkeys suggested the same trend [9]. In our study, despite the PCR tests being positive, the viral culture was negative in all tested specimens. Furthermore, all the HCP who did not wear recommended personal protective equipment showed no signs of infection such as fever and respiratory symptoms, and all the results of nasopharyngeal swab PCR tests were negative. Thus, the hypothesis that a patient with re-positive PCR is not infectious is plausible. However, it is easy to say that culture positive means that the samples are infectious, but it is important to note that culture negative cannot be said to be non-infectious [10]. Moreover, there are many facts about this virus that are yet unknown, and it should still be necessary to maintain infection control policies from the viewpoint of preventing the spread of infection especially in medical institutions.

### Conclusion

In conclusion, no HCP were infected by contact with and aerosol exposures to SARS-CoV-2 re-positive patients in our hospital.

## Limitations

There were several limitations to this study. First, the sample size was small. However, we investigated not only PCR test but viral culture for the index patient. And we also investigated PCR tests, and antibodies twice for the exposed HCP. Second, there were lack of information about degree or duration of aerosol exposures. Thus, further investigation is required into whether SARS-CoV-2 re-positive patients are infectious.

## Data Availability

All data generated or analyzed during this study are included in this published article.

## Abbreviations

SARS-CoV-2: Severe acute respiratory syndrome coronavirus 2
COVID-19: Coronavirus disease SARS-CoV-2
PCR: Polymerase chain reaction
HCP: Healthcare personnel
CDC: Centers for Disease Control and Prevention
ICU: Intensive care unit
IgG: Immunoglobulin G
